# Impact of swine influenza A virus on porcine reproductive and respiratory syndrome virus infection in alveolar macrophages

**DOI:** 10.3389/fvets.2024.1454762

**Published:** 2024-08-26

**Authors:** Janaïna Grevelinger, Olivier Bourry, François Meurens, Aline Perrin, Caroline Hervet, Laurence Dubreil, Gaëlle Simon, Nicolas Bertho

**Affiliations:** ^1^Oniris, INRAE, BIOEPAR, Nantes, France; ^2^ANSES, Ploufragan-Plouzané-Niort Laboratory, Swine Virology Immunology Unit, Ploufragan, France; ^3^CRIPA, Fonds de Recherche du Québec, Département de pathologie et microbiologie, Faculté de médecine vétérinaire, Université de Montréal, Saint-Hyacinthe, QC, Canada; ^4^Oniris, INRAE, APEX, PAnTher, Nantes, France

**Keywords:** co-inoculation, co-infection, porcine respiratory disease complex, interferon, lung

## Abstract

Porcine respiratory disease complex represents a major challenge for the swine industry, with swine influenza A virus (swIAV) and porcine reproductive and respiratory syndrome virus (PRRSV) being major contributors. Epidemiological studies have confirmed the co-circulation of these viruses in pig herds, making swIAV-PRRSV co-infections expected. A couple of *in vivo* co-infection studies have reported replication interferences between these two viruses. Herein, using a reductionist *in vitro* model, we investigated the potential mechanisms of these *in vivo* interferences. We first examined the impact of swIAV on porcine alveolar macrophages (AMs) and its effects on AMs co-infection by PRRSV. This was done either in monoculture or in co-culture with respiratory tracheal epithelial cells to represent the complexity of the interactions between the viruses and their respective target cells (epithelial cells for swIAV and AMs for PRRSV). AMs were obtained either from conventional or specific pathogen-free (SPF) pigs. SwIAV replication was abortive in AMs, inducing cell death at high multiplicity of infections. In AMs from three out of four conventional animals, swIAV showed no impact on PRRSV replication. However, inhibition of PRRSV multiplication was observed in AMs from one animal, accompanied by an early increase in the expression of interferon (IFN)-I and IFN-stimulated genes. In AMs from six SPF pigs, swIAV inhibited PRRSV replication in all animals, with an early induction of antiviral genes. Co-culture experiments involving tracheal epithelial cells and AMs from either SPF or conventional pigs all showed swIAV-induced inhibition of PRRSV replication, together with early induction of antiviral genes. These findings highlight the complex interactions between swIAV and PRRSV in porcine AMs, and would suggest a role of host factors, such as sanitary status, in modulating viral propagation. Our co-culture experiments demonstrated that swIAV inhibits PRRSV replication more effectively in the presence of respiratory tracheal epithelial cells, suggesting a synergistic antiviral response between AMs and epithelial cells, consistent with *in vivo* experiments.

## 1 Introduction

Porcine Respiratory Disease Complex (PRDC) is a multifactorial syndrome that leads to substantial losses in the swine industry (1, 2). It is caused by the association of various pathogens, including viruses and bacteria, which can persist in the host and circulate simultaneously within a herd. Common agents causing PRDC include swine influenza A virus (swIAV), porcine reproductive and respiratory syndrome virus (PRRSV), porcine circovirus type 2 (PCV2), and *Mycoplasma hyopneumoniae* (Mhp). Epidemiological studies have identified a noteworthy association between porcine swIAV and PRRSV, underlining the importance of investigating their interactions (1, 3–6).

PRRSV, classified as a member of the *Arteriviridae* family within the *Nidovirales* order, is an enveloped virus with a positive-single-stranded RNA genome. PRRSV is categorized into two distinct species: *Betaarterivirus suid* 1 (PRRSV-1), primarily found in Europe, and *Betaarterivirus suid 2* (PRRSV-2), mostly prevalent in North America and Asia (7, 8). The virus induces long-term infections characterized by interference with type I interferon induction (9–13).

Swine IAV belongs to the genus *Alphainfluenzavirus* within the *Orthomyxoviridae* family. It is an enveloped virus with a negative-single-stranded, segmented RNA genome. The most commonly observed subtypes of influenza A viruses in pigs worldwide are H1N1, H1N2, and H3N2 (14). Unlike PRRSV, swIAV leads to acute respiratory disease and the induction of high levels of type I interferon (15–17).

Given the worldwide prevalence of both PRRSV and swIAV in the large majority of swine rearing regions, there is a significant risk of simultaneous infections in pig herds. Therefore, investigating the interaction between these two viruses is important for a comprehensive understanding of PRDC.

Macrophages play an essential role in the initial defense against respiratory infections through their functions of phagocytosis and professional antigen-presenting cells. They also contribute to homeostasis by clearing apoptotic cells, secreting tissue repair factors, and participating in tissue remodeling (18, 19). PRRSV infects cells from the monocyte lineage, with porcine alveolar macrophages (AMs) considered to be the main target cells (15). During PRRSV infection, the virus alters AMs function by disrupting cytokine production and inducing apoptosis (15, 20). SwIAV preferentially replicates in the epithelial cells of pig respiratory tracts, but AMs may also be susceptible to the virus (21–23). Regarding the impact of swIAV on AMs, studies have primarily focused on the virus’ ability to replicate within these cells. It is recognized that influenza A virus (IAV) replication in macrophages is influenced by both the viral strain and the type of macrophages. The most compelling evidence of IAV replication in macrophages has been demonstrated in macrophages from mice and humans, originating from studies focusing on the highly pathogenic avian H5N1 virus (24–26). However, further research is needed to better understand the interactions between other swIAV (which are not highly pathogenic) and AMs in pigs, particularly as swIAV infections are frequently associated with other respiratory pathogens.

SwIAV and PRRSV co-infections have been studied more thoroughly *in vivo* than *in vitro* (5, 6)*. In vivo*, the clinical results of PRRSV/swIAV superinfections vary according to the experimental conditions and the virus strains studied. While some studies reported exacerbations of lung lesions (27, 28), others showed no difference in clinical signs compared with animals infected with a single virus (29–31). Additionally, the two viruses can interfere with each other (17). These contradictory data advocate for more reductionist *in vitro* studies on PRRSV/swIAV co-infections.

The effects of swIAV/PRRSV co-infections at the cellular and molecular levels are still poorly understood. *In vitro* studies are important for understanding the direct interactions between viruses and specific cell types. This approach is essential for dissecting viral mechanisms and cellular immune responses in a controlled environment, but such *in vitro* studies have been less explored for co-infections with swIAV and PRRSV.

An *in vitro* study on porcine tracheal epithelial cells have shown that, although PRRSV does not infect epithelial cells, it can modify the cells’ interferon response when inoculated simultaneously or shortly before with swIAV, thereby hindering the production of swIAV (32). Interestingly, a study using conventional pig lung tissue sections and AMs, the target cells of PRRSV, present results suggesting that in some conditions, upon co-infection, swIAV may inhibit PRRSV-2 replication (33). Whether this inhibition occurs also for PRRSV-1 strains, and what is the mechanisms and kinetic of this process remain to be explored.

In this work, we assessed the effects of swIAV on AMs and evaluated the impact of swIAV and PRRSV-1 mono-or co-inoculation on AMs, as well as on the host cells targeted by both types of viruses, within a respiratory epithelial cells/AMs co-culture system. Additionally, this study employs a kinetic approach to elucidate potential interaction between the viruses by monitoring infection progression over time to identify critical phases and interferences in viral replication. Notably, the study also compares AMs sampled from animals from two rearing facilities presenting different sanitary status.

In this study, we evaluated the interactions between a swIAV strain belonging to the European human-like reassortant swine H1N2 lineage and a PRRSV strain from the PRRSV-1 subtype 1, two viruses that were co-circulating in pig farms in Brittany, France, and for which epidemiological investigations reported a significant association regarding seropositive status (34).

## 2 Materials and methods

### 2.1 Virus production and titration

The swIAV strain A/swine/Ille et Vilaine/0415/2011 (H1_hu_N2), selected from the repository of the French National Reference Laboratory for Swine Influenza (ANSES, Ploufragan, France), was isolated in Brittany, France, from a pig with acute respiratory syndrome. The virus was propagated on Madin-Darby Canine Kidney (MDCK) (ATCC reference CCL-34) cells for 24 hours (h) in Dulbecco’s Modified Eagle Medium (DMEM) (Eurobio scientific, Les Ulis, France) supplemented with 10% fetal calf serum (FCS) (Eurobio scientific) and 1% Penicillin (100 UI/mL)-Streptomycin (100 μg/mL) (PS) (Eurobio scientific), and 2 μg/mL of trypsin TPCK treated (Worthington Biochemical Corp., Lakewood, NJ, United States). Following collection, the supernatant underwent clarification by centrifugation (600 × g, 20 min) and subsequent purification on Amicon Ultra-15 Centrifugal Filters (Sigma-Aldrich) after a 30 min centrifugation at 4,000 × g and 4°C. Virus titer was determined using the Tissue Culture Infectious Dose (TCID_50_) assay protocol on MDCK and calculated according to the Reed and Muench method (35). The final titer of the viral stock reached 10^8^ TCID_50_/mL.

The PRRSV strain PRRS-FR-2005-29-24-1 (Finistère strain; PRRSV-1 subtype 1) (ANSES’ collection) was isolated in Brittany, France, in 2005 from a pig herd whose sows had experienced reproductive failures. This strain is identified as low pathogenic (36). The virus was cultured on fresh porcine alveolar macrophages (AMs) in Roswell Park Memorial Institute (RPMI) 1640 medium (Eurobio scientific) supplemented with 10% FCS and 1% Penicillin (100 UI/mL)-Streptomycin (100 μg/mL)-Amphotericin (0.25 μg/mL) (PSA) (Dutscher) for 48 h after isolation. After clarification and purification using Amicon filters, the virus titer was determined using the (TCID_50_)protocol on AMs and calculated using the Kärber method (37). The resulting viral titer was 10^9^ TCID_50_/mL.

### 2.2 Alveolar macrophages

AMs were sourced from five 5-to 6-month-old conventional pigs of the Large White breed. These pigs were euthanized as part of routine management procedures at the *Unité Expérimentale de Physiologie Animale de l’Orfrasière* (UEPAO, Tours, France). They were serologically free of PRRSV, and their status regarding swIAV is unknown. Collection of AMs was performed through two bronchoalveolar lavages (BAL) on the lungs, utilizing 250 mL phosphate-buffered saline (PBS) supplemented with 2 mM ethylenediaminetetraacetic acid (EDTA) (Sigma-Aldrich) for each lavage.

Similarly, AMs from six 8-week-old specific pathogen-free (SPF) piglets were acquired through bronchoalveolar lavages. These piglets were obtained from the SPF herd (Supplementary Table S1) of the French Agency for Food, Environmental and Occupational Health & Safety (ANSES, Ploufragan, France).

The obtained AMs were frozen in FCS with 10% DMSO and stored in liquid nitrogen.

### 2.3 Newborn pig tracheal epithelial cell line

NPTr cells (38) were cultured in Minimum Essential Medium (MEM, Eurobio scientific) supplemented with 10% FCS and 1% PSA, and incubated at 37°C in 5% CO_2_.

### 2.4 Cell cultures and virus inoculations

For the AMs monocultures, cells were thawed, seeded in 24-well plates at 1.6 × 10^6^ cells per well. After 1 h of incubation, they were mixed by pipetting, followed by another hour of incubation. After the 2 h incubation period, non-adherent cells were removed by washing twice with RPMI-1640 medium, enriching the BAL cells with adherent AMs.

After 2 h of incubation following thawing, AMs cultures were single or co-inoculated with swIAV and PRRSV, at a multiplicity of infection (MOI) of 0.5 or 1, respectively. For co-inoculation, both viruses were mixed extemporaneously, immediately prior to be deposited on cells. Cells cultured in complete RPMI medium only served as negative controls. Virus adsorption was allowed for 1 h at 37°C and 5% CO_2_ with rocking agitation. Subsequently, the cultured cells were washed with RPMI and maintained at 37°C and 5% CO_2_ in 1 mL of RPMI medium supplemented with 1% PS and 10% FCS. At 1, 12, 24, 36, and 48 h post-inoculation (hpi) cell supernatants were collected and stored at −80°C. The cells were lysed using the lysis buffer of the NucleoSpin RNA Plus XS kit (Macherey-Nagel, Düren, Germany), and stored at −80°C.

For the NPTr/AMs co-cultures, AMs and NPTr cells were cultured in MEM supplemented with 10% FCS and 1% PS, and incubated at 37°C in 5% CO_2_. NPTr cells were seeded in 24-well plates at a density of 3 × 10^5^ cells per well and cultured at 37°C and 5% CO_2_ for 24 h to reach 100% confluence. After washing with MEM, AMs were added to the culture at 1.2 × 10^6^ cells per well and incubated at 37°C and 5% CO_2_ for 24 h to promote their adherence to NPTr cells. NPTr/AMs co-cultures were inoculated with viruses in the same manner as the AMs monoculture protocol, using either single or co-inoculation with swIAV at an MOI of 0.5 and PRRSV at an MOI of 1. The MOI was determined by counting the total number of cells in the NPTr/AMs co-culture wells just before virus inoculation. After the virus inoculation step, co-cultured cells were kept at 37°C with 5% CO_2_ in 2 mL of complete MEM medium. Cell lysates and supernatants were collected and stored at −80°C at 1, 12, 24, and 48 hpi. The cell layers were lysed using the lysis buffer of the RNeasy Mini Kit (Qiagen), and stored at −80°C.

### 2.5 Cell viability assays

Conventional pig AMs were thawed and seeded at 1.2 × 10^6^ cells per well in an 8-well μ-Slide plate (Cat. No. 80826, ibidi GmbH, Germany) using complete RPMI medium. The cells were inoculated for 48 h with swIAV at different MOI 0.1, 0.5, and 2, or with PRRSV at a MOI of 2 to serve as a positive control for cell death. Subsequently, the cells were stained with Hoechst 33342 (Sigma-Aldrich, 14,533, bisBenzimide H 33342 trihydrochloride) at 5 μg/mL for 10 min at 37°C, followed by staining with Propidium Iodide (PI) (Sigma, P4170) at a final concentration of 1.5 × 10^−3^ mM for an additional 10 min at room temperature (RT). The labeled cells were then fixed with 1% paraformaldehyde (PFA) for 15 min at 37°C, followed by a PBS rinse. Fluorescence from labeled cells was analyzed using Confocal laser scanning microscopy (CSLM) (Zeiss LSM 780, Germany) with Plan Apochromat 20x/0.8NA objective lens. Dual sequential laser excitation with 405 nm and 561 nm beam laser was used to observe both fluorescent signals generated by Hoechst (405 nm excitation) and Propidium Iodide (561 nm excitation). The images were analyzed, and the cells were counted using Fiji software (39).

### 2.6 Immunofluorescence assays

In an 8-well μ-Slide plate, NPTr cells were seeded at 6 × 10^4^ cells per well and cultured with complete MEM at 37°C and 5% CO_2_ for 24 h. Conventional pig AMs were thawed and seeded at 1.6 × 10^6^ cells per well using complete RPMI medium at 37°C and 5% CO_2_ overnight. The cells were inoculated with swIAV for 6 and 12 h at MOI 0.5 for each type of cell.

The cells were fixed with 1% PFA for 10 min at RT and permeabilized with 0.2% Triton X-100 (Sigma-Aldrich) for an extra 10 min. SwIAV infected cells were identified using a mouse monoclonal antibody targeting the viral nucleoprotein (dilution 1/50) (OBT0846, clone: 1341, Bio-Rad), and cell nuclei were stained with 4′,6′-diamidino-2-phenylindole (DAPI) (Sigma-Aldrich) at 2 μg/mL for 30 min at RT in a PBS-5% goat serum-5% pig serum blocking solution. This step was followed by incubation with an appropriate goat anti-mouse secondary antibody coupled to Alexafluor555 (dilution 1/100) (Ref: A21121, Invitrogen) for 30 min at RT in the same blocking solution. Washes between each step were performed with PBS-0.5% Tween 20 (Sigma-Aldrich). The cells were then fixed again with 1% PFA. Fluorescence from labeled cells was analyzed using CSLM (Nikon C2, Nikon Europe B.V., Amsterdam, Netherlands) with APO LWD 40x/1.15NA water immersion objective lens. Dual sequential laser excitation with 405 nm and 561 nm beam laser was used to observe both fluorescent signals generating by DAPI (405 nm excitation) and Alexafluo555 (561 nm excitation). The images were analyzed, and the cells were counted using Fiji software (39).

### 2.7 Immune gene expression analysis and viral quantification by quantitative reverse transcription polymerase chain reaction

Total RNA extraction from AMs cultures and NPTr/AMs co-cultures was performed using the NucleoSpin RNA Plus XS kit (Macherey-Nagel, Düren, Germany) and the RNeasy Mini Kit (Qiagen), respectively, following the manufacturer’s instructions. RNA from cell culture supernatants was extracted using the NucleoSpin Virus Mini kit for viral RNA/DNA purification (Macherey-Nagel, Düren, Germany), following the manufacturer’s instructions.

The quantity and quality of the total RNA were assessed using the Nanophotometer (Implen, Munich, Germany). Subsequent cDNA synthesis was performed using reverse transcriptase in the iScript Reverse Transcription Supermix for RT-qPCR (Bio-Rad, Hercules, CA, United States). The resulting cDNA was then combined with primer/probe sets and IQ SYBR Green Supermix (Bio-Rad), in accordance with the manufacturer’s recommendations. The qPCR assays replicated the methods described in a previous study (32) (Supplementary Table S2).

Viral genome quantification was performed through semi-quantitative reverse transcription-polymerase chain reaction using TaqMan technology with Takyon No Rox Probe MasterMix dTTP blue 2× (Eurogentec, Liège, Belgium). The targeted regions included the open reading frame 5 (ORF5) of the Finistère strain, and the M gene of swine influenza A virus following the methodology described by Saade et al. (32).

The qPCR assays were conducted on a CFX96 Connect and CFX Opus (Bio-Rad). For internal normalization, samples were simultaneously evaluated using the average Cycle quantification (Cq) of two stable reference genes in each sample, namely, the ribosomal protein L19 (RPL19) and the ribosomal protein S24 (RPS24) (40). Subsequently, qPCR data (Cq) underwent Genex macro analysis (Bio-Rad) (41) and were expressed as relative values following the Genex macro analysis.

### 2.8 Statistics

Due to the non-normal distribution, tested by Shapiro–Wilk test, the Mann–Whitney unpaired test was used and the Kruskal-Wallis test was employed to analyze cellular gene expression. These analyses were performed using GraphPad Prism (GraphPad Software version 9.5.1, San Diego, CA, United States).

The number of animals used and the replicates are specified in the legends of each figure.

Virus titers below the detection limit (2.1 log_10_ TCID_50_/mL) were assigned a value of 0 log_10_ TCID_50_/mL.

## 3 Results

### 3.1 swIAV inoculation on alveolar macrophages from conventional pigs

**Figure 1:**
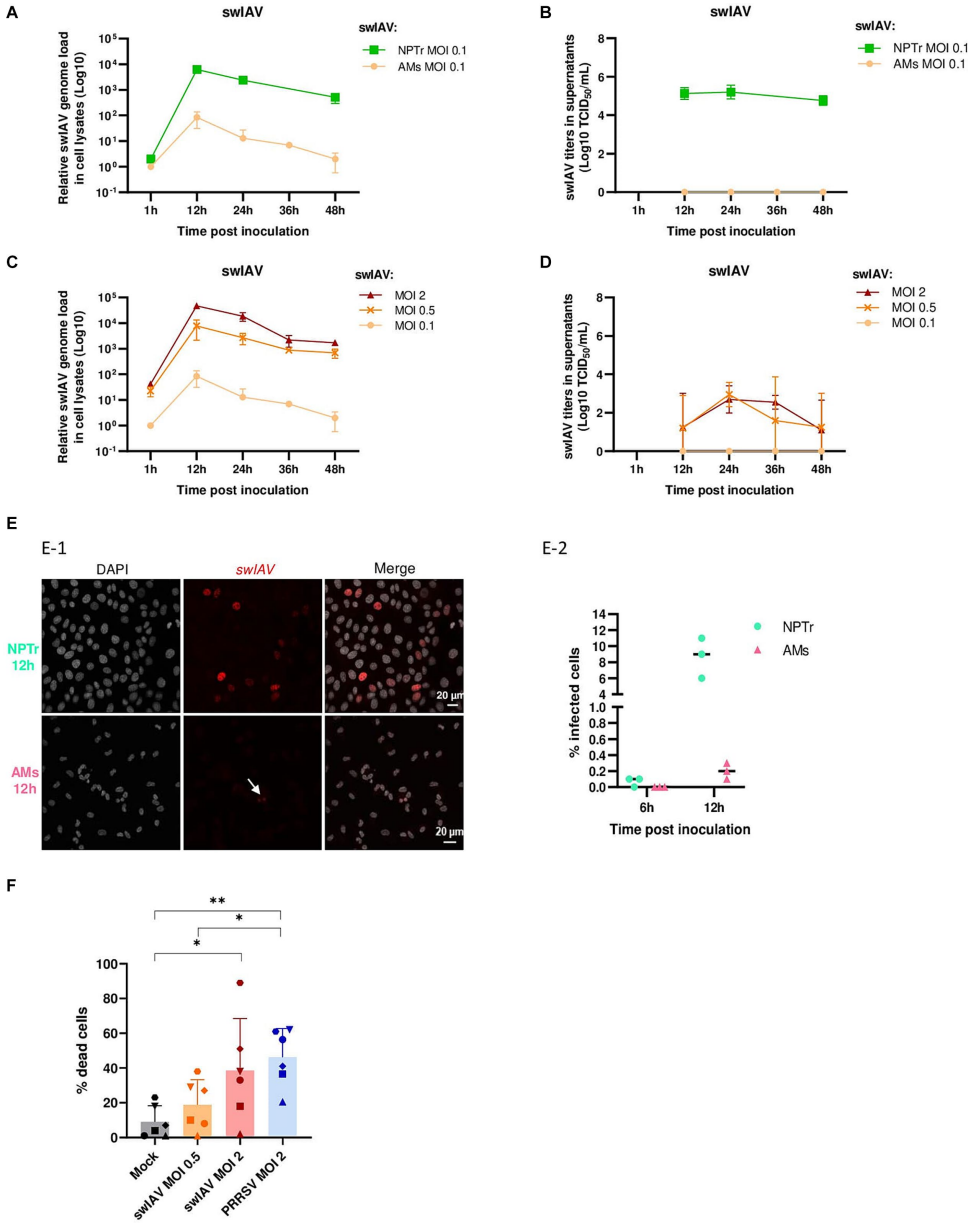
Inoculation of swine influenza A virus on conventional porcine alveolar macrophages. **(A,B)** Conventional porcine AMs and NPTr were inoculated with swIAV at MOI 0.1. Adherent cells and supernatants were sampled at different times post-inoculation (1, 12, 24, 36, 48 h). Expression of the swIAV viral genome was quantified by RT-qPCR **(A)**, and the virus was titrated in supernatants **(B)** (mean ± SD; for AMs *n* = 2, and for NPTr *n* = 3). **(C,D)** Conventional porcine AMs were inoculated with swIAV at different MOI 0.1, 0.5, and 2. Adherent cells and supernatants were sampled at different times post-inoculation (1, 12, 24, 36, 48 h). Expression of the swIAV viral genome was quantified by RT-qPCR **(C)**, and the virus was titrated in supernatants **(D)** (mean ± SD; *n* = 2). **(E)** AMs and NPTr cells were inoculated with swIAV at a MOI of 0.5. Cells were fixed after 6 h and 12 h post-inoculation and stained with an antibody against the nucleoprotein to detect infected cells (in red). Cell nuclei were stained (in white) using 4′,6′-diamidino-2-phenylindole (DAPI). **(E1)** Confocal imaging of NPTr and AMs cells, 12 h post inoculation with swIAV. Upper panel NPTr cells. Lower panel AMs cells. Nuclear staining with DAPI, swIAV immunodetection and merge (from left to right). Scale bars = 20 μm. **(E2)** The number of infected cells was calculated in relation to the total number of cells (*n* = 3, technical replicate). **(F)** AMs were inoculated with either medium alone or swIAV at a MOI of 0.5 or 2; or PRRSV at a MOI of 2. After 48 h, cells were stained with propidium iodide/Hoechst. Each symbol corresponds to AMs from a single animal. The number of dead cells was calculated in relation to the total number of cells. Statistics Mann–Whitney unpaired, non-parametric test, (*) *p* < 0.05 or (**) *p* < 0.01 (mean ± SD; *n* = 6).

We first compared the ability of AMs and epithelial cells to support swIAV gene expression and particle production using a low MOI of 0.1 at different post-inoculation (pi) times: 1, 12, 24, 36, and 48 h. In both AMs and NPTr cells, intracellular viral gene expression peaked at 12 hpi and then decreased over time. However, at 12 hpi, the viral genome was expressed 74 times more in NPTr cells than in AMs (Figure 1A). Then, titration of infectious viral particles in cell culture supernatants was performed, and no infectious virus was detected for AMs. In contrast, in NPTr cells, which served as a positive control for viral replication, swIAV inoculated at a MOI of 0.1 showed productive replication, with high replication remaining constant between 12 and 48 h and average titers of 10^5.03^ TCID_50_/mL (Figure 1B). Interestingly, when higher MOIs of 0.5 and 2 were used, viral genome expression in AMs increased in a MOI-dependent manner (Figure 1C). However, virus titration revealed only low titers that peaked at 24 h, averaging 10^2.95^ and 10^2.70^ TCID_50_/mL for MOIs of 0.5 and 2, respectively (Figure 1D).

Microscopic analysis of MOI 0.5 inoculated cells at 6 and 12 hpi confirmed that swIAV did not undergo productive replication in AMs. Indeed, at 6 hpi, an average of 0.1% of NPTr cells were positive for swIAV nucleoprotein staining, while no AMs were detected as positive for swIAV nucleoprotein. By 12 hpi, the rate of positive cells increased to an average of 8% in NPTr cells and 0.2% in AMs (Figure 1E).

The cytotoxic effect of swIAV on host cells was evaluated using PI/Hoechst staining. AMs were inoculated with swIAV at MOIs 0.5 and 2, or with PRRSV at MOI 2, used as a positive control to induce AMs death. Following a 48 h swIAV exposure, a significant (*p* < 0.05) number of dead cells were observed at MOI 2 but not at MOI 0.5 (Figure 1F).

Thus, in order to study the impact of swIAV on PRRSV multiplication in AMs, we subsequently inoculated swIAV at MOI 0.5, the highest concentration that did not induce significant AMs cell death.

### 3.2 Co-inoculation of swIAV and PRRSV on alveolar macrophages from conventional pigs

**Figure 2:**
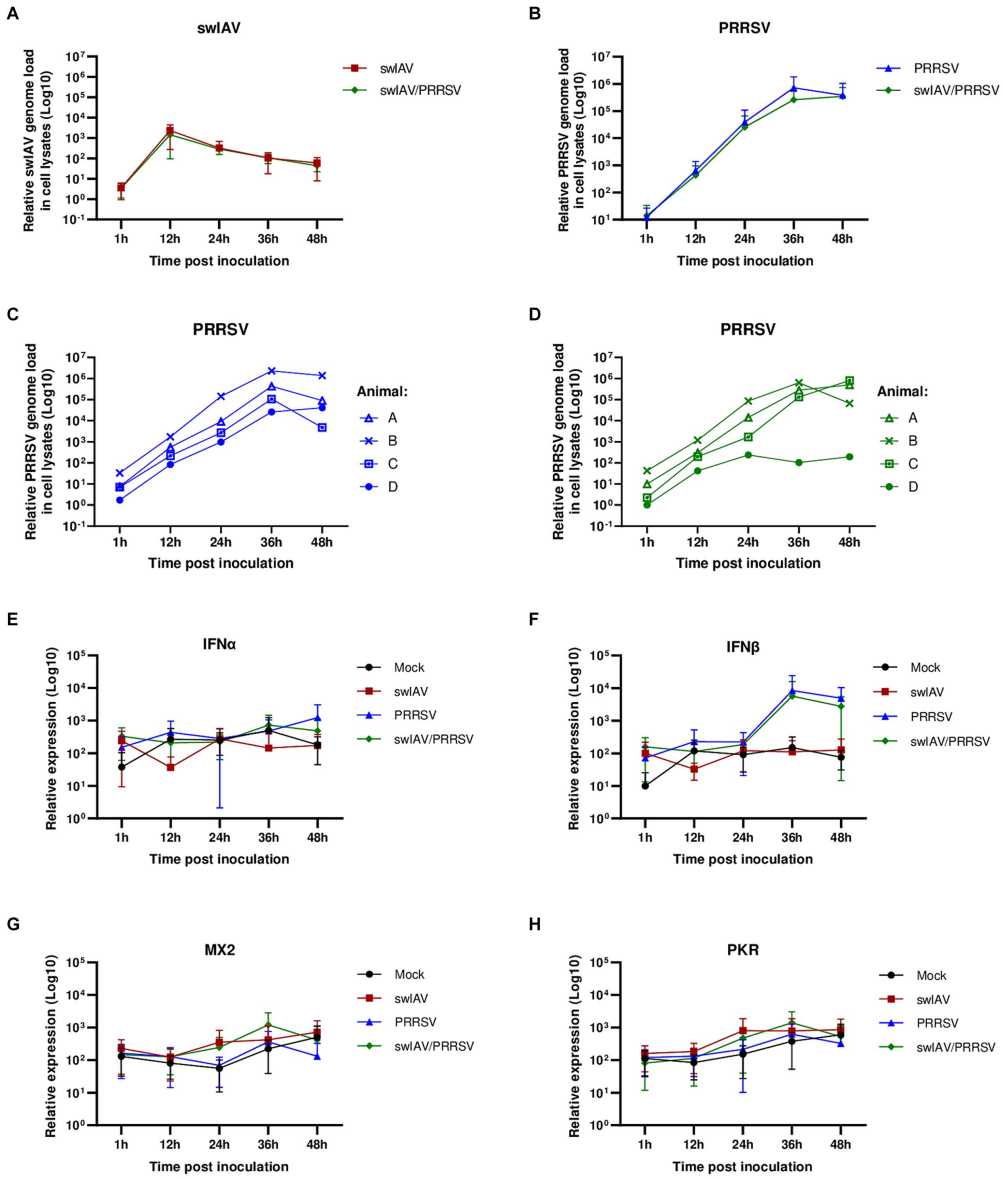
Simultaneous co-inoculation of swIAV and PRRSV viruses on AMs from conventional pigs. AMs were inoculated with either culture medium, swIAV at a MOI of 0.5, PRRSV at a MOI of 1, or both viruses simultaneously. Samples were collected at 1, 12, 24, 36 and 48 h. **(A,B)** The expression of the swIAV **(A)** and PRRSV **(B)** viral genomes was quantified using RT-qPCR in cell lysates (mean ± SD; *n* = 4). **(C,D)** For the PRRSV inoculated and swIAV/PRRSV co-inoculated conditions, the expression of the PRRSV genome was individually evaluated for AMs from each animal. **(E–H)** The expression of the antiviral genes IFNα, IFNβ, MX2, and PKR were analyzed by RT-qPCR. Statistics Kruskal-Walis, non-parametric test (mean ± SD; *n* = 4). Data are combined from 2 independent experiments.

To explore how swIAV infection might affect interactions between AMs and PRRSV, we co-inoculated AMs with swIAV and PRRSV. PRRSV was used at the intermediate MOI of 1 that would allow a strong infectious input while providing space for further viral amplification. AMs from conventional pigs were exposed to culture medium, to swIAV (MOI 0.5), to PRRSV (MOI 1), or to both viruses simultaneously at MOI 0.5 and 1, respectively. Samples were collected at 1, 12, 24, 36, and 48 hpi, and the expression of swIAV and PRRSV transcripts were analyzed (Figures 2A–D).

The results confirmed that swIAV did not replicate in AMs, and co-inoculation with PRRSV did not affect the replication of swIAV in AMs (Figure 2A). As expected, PRRSV inoculation of AMs led to strong viral amplification, regardless of the animal from which the cells originated (Figures 2B,C). In swIAV/PRRSV co-inoculation condition, the PRRSV genome replicated similarly to when PRRSV was inoculated alone in AMs from only three out of four animals (Figure 2D). However, in AMs from one animal (animal *D*), we observed an inhibition of PRRSV replication starting between 24 and 36 h post-inoculation, suggesting that swIAV co-inoculation had an impact on PRRSV replication in this specific case (Figure 2D).

Thus, the expression of antiviral genes was analyzed (Figures 2E–H). Interferon levels exhibited considerable variability among different conditions and among individuals. No difference was observed in interferon alpha (IFNα) levels across conditions and over time (Figure 2E). However, for interferon beta (IFNβ), in the conditions inoculated with PRRSV (PRRSV and swIAV/PRRSV groups), a trend toward increased IFNβ expression at 36 h was observed, although it was not significant (*p* > 0.05) compared to the Mock and swIAV groups (Figure 2F). Additionally, the expression of interferon-stimulated genes (ISGs) MX2 and PKR was examined (Figures 2G,H). As indicated by their name, ISG expression is triggered in response to interferon production and is thus indicative of actual protein expression and the release of type I and III interferons (42). Similar to interferons, variability was also observed in ISG expression (Supplementary Figure S1). Interestingly, despite IFNβ expression appears to be increased in the PRRSV and swIAV/PRRSV groups as early as 36 h, ISGs were not induced (Figures 2G,H). Upon observing the expression of ISGs for each individual, we noted a notable change at 12 hpi for animal *D* under conditions infected with the swIAV virus (Supplementary Figure S1). At this time point, we observed that only in animal *D*, MX2 and PKR showed higher expression under exposure to swIAV and swIAV/PRRSV compared to the Mock and PRRSV groups, unlike the other animals (Supplementary Figures S2A,B).

### 3.3 Co-inoculation of swIAV and PRRSV on alveolar macrophages from specific pathogen-free piglets

**Figure 3:**
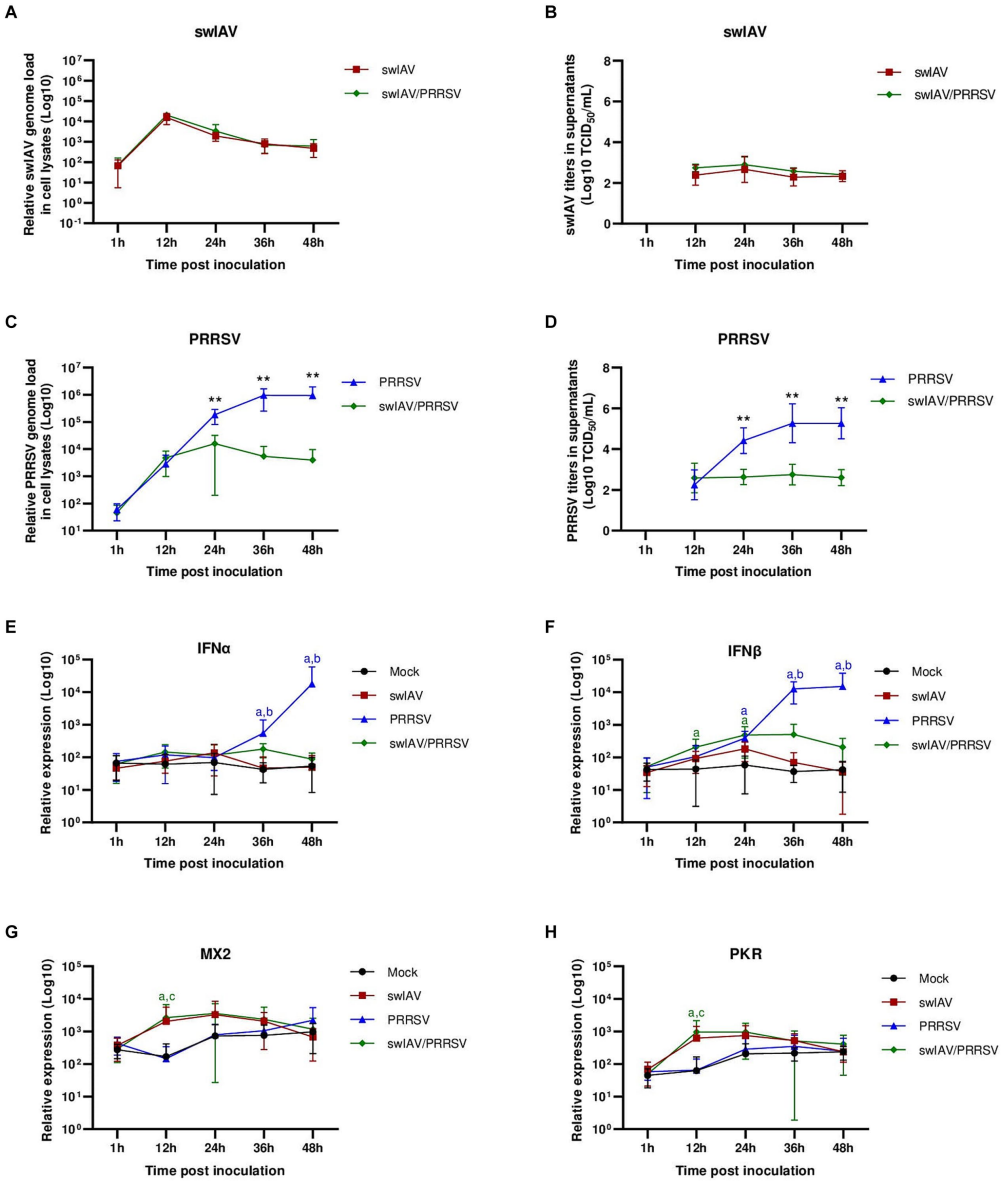
Simultaneous co-inoculation of swIAV and PRRSV viruses on AMs from specific pathogen-free piglets. AMs were inoculated with either culture medium, swIAV at a MOI of 0.5, PRRSV at a MOI of 1, or both viruses simultaneously. Samples were collected at 1, 12, 24, 36 and 48 h. **(A–D)** The expression of swIAV viral genome was quantified in the cell lysates using RT-qPCR **(A)**, and the virus was titrated in supernatants **(B)**. The expression of PRRSV viral genome was quantified in the cell lysates using RT-qPCR **(C)**, and the virus was titrated in supernatants **(D)**. Statistics Mann–Whitney unpaired, non-parametric test, (*) *p* < 0.05 or (**) *p* < 0.01 (mean ± SD; *n* = 5–6). **(E–H)** The expression of the antiviral genes IFNα, IFNβ, MX2 and PKR were analyzed by RT-qPCR. Statistics Kruskal-Walis, non-parametric test. Different letters (a–d) indicate that the considered group (specified by its color) was significantly different from the Mock group (a), from the swIAV group (b), from the PRRSV group (c) or from the swIAV/PRRSV group (d) with *p* < 0.05 (mean ± SD; *n* = 6). Data are combined from 2 independent experiments.

We hypothesized that the variability in ISG induction during swIAV interaction depending on AMs donor could be attributed to animal history of lung inflammation like environmental particles (43), or former contacts with infectious stimuli, as described previously in murine and human studies, which indicated a modification in the AMs response upon viral (44) or bacterial (45) stimulations. Therefore, we aimed to investigate the direct effects of swIAV and PRRSV on AMs while minimizing external factors like previous infections, which could potentially influence the macrophage response. In order to investigate this possibility, we sought to replicate these experiments using AMs from younger animals, thus exposed for a shorter time to a potentially inflammatory environment, originated from a specific pathogen-free (SPF) herd bred under air filtration (Figures 3A–H).

Following swIAV inoculation and swIAV/PRRSV co-inoculation, we observed similar swIAV replication as observed in conventional AMs, further confirming that swIAV underwent abortive replication in porcine AMs (Figures 3A,B). However, concerning PRRSV, co-inoculation with swIAV led to an inhibition of PRRSV genome replication from 24 hpi, paralleled with an inhibition of infectious viral particle production (Figures 3C,D; Supplementary Figure S3).

Next, we assessed the expression of antiviral genes (Figures 3E–H). For the swIAV inoculation condition, no difference in interferon expression was observed (Figures 3E,F). However, there was a slight, non-significant increase in ISG MX2 and PKR at 12 hpi (Figures 3G,H). Upon co-inoculation with swIAV/PRRSV, an early significant (*p* < 0.05) increase in IFNβ transcriptomic expression was noted at 12 hpi (Figure 3F). This increase in IFNβ was accompanied by increases in MX2 and PKR ISG expression (*p* < 0.05) (Figures 3G,H). In the PRRSV condition, significant (p < 0.05) IFNβ and IFNα expressions were observed at later times post-infection, respectively 24 and 36 hpi (Figures 3E,F). However, this transcriptomal type-I IFN increase did not result in functional IFN-I protein release since no significant (*p* > 0.05) increase in ISG expression was observed compared to the control condition (Figures 3G,H).

These results suggested that exposure to swIAV in AMs from SPF pigs quickly activated the expression of antiviral genes, potentially playing a role in inhibiting PRRSV replication.

### 3.4 Co-inoculation of swIAV and PRRSV in an epithelial respiratory cells/alveolar macrophages co-culture system

**Figure 4:**
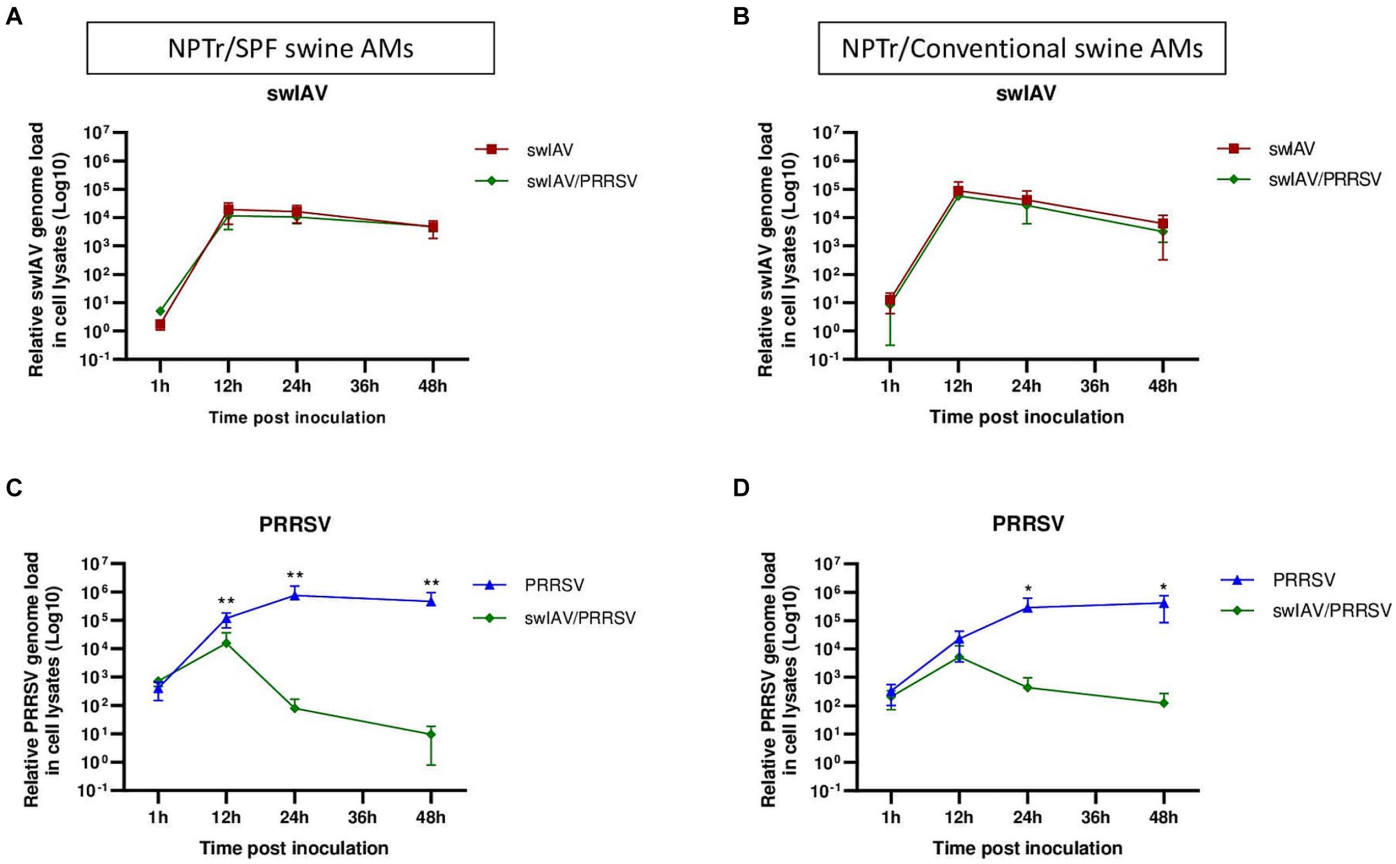
Simultaneous co-inoculation of swIAV and PRRSV viruses on epithelial cell line/AMs co-cultures derived from both specific pathogen-free and conventional swine. NPTr epithelial cells and AMs were inoculated with either culture medium, swIAV at a MOI of 0.5, PRRSV at a MOI of 1, or both viruses simultaneously. Samples were collected at 1, 12, 24, and 48 h. On the left side are presented the results for SPF pig AMs, and on the right are the results for conventional pig AMs. **(A,B)** The expression of the swIAV viral genome was quantified in the cell lysates using RT-qPCR. **(C,D)** The expression of the PRRSV viral genome was quantified in the cell lysates using RT-qPCR. Statistics Mann–Whitney unpaired, non-parametric test, (*) *p* < 0.05 or (**) *p* < 0.01 (mean ± SD; *n* = 6 for SPF swine AMs; *n* = 4 for conventional swine AMs). Data are combined from 2 independent experiments for AMs originating from SPF or conventional swine.

**Figure 5:**
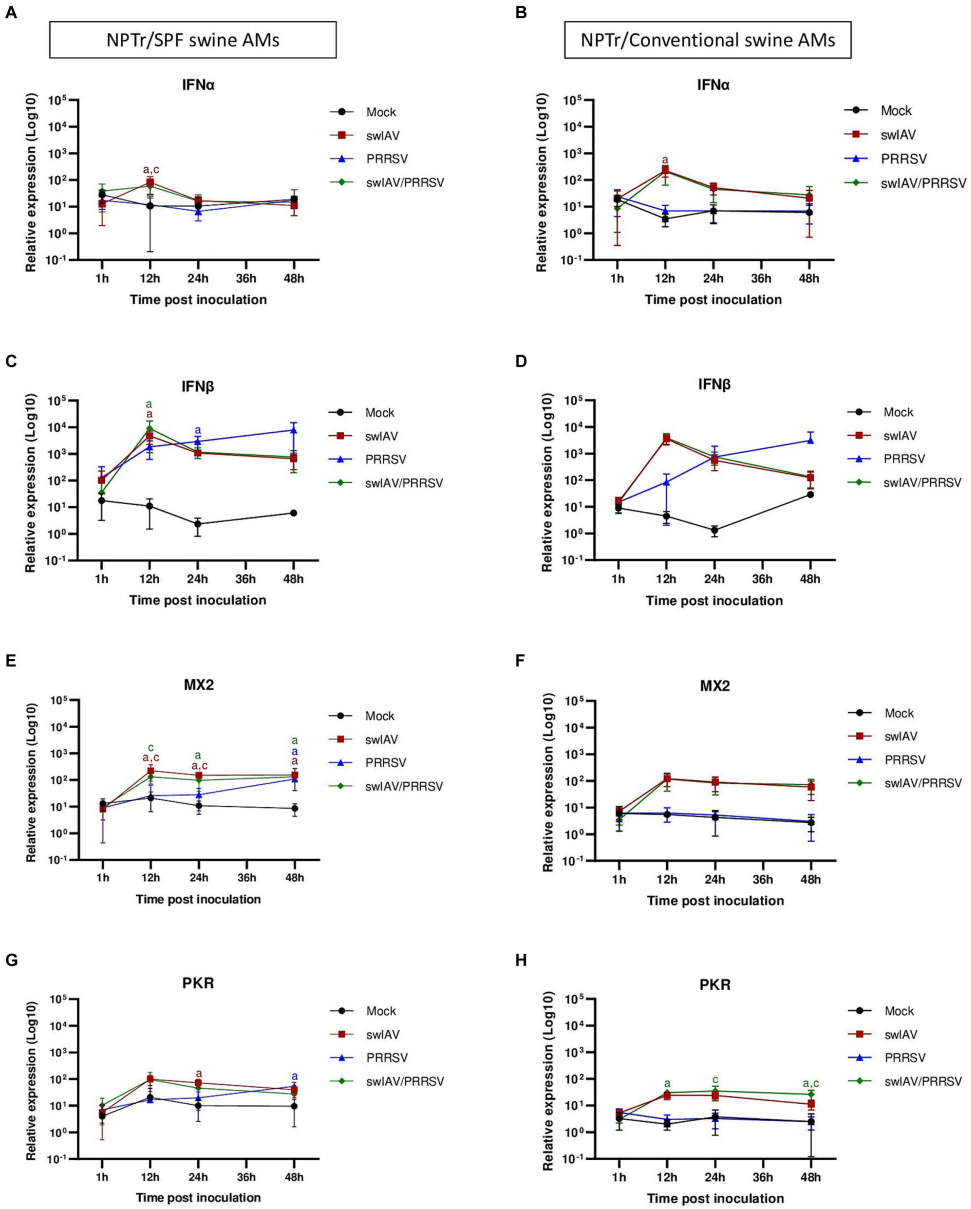
Simultaneous co-inoculation of swIAV and PRRSV viruses on epithelial cell line/AMs co-cultures derived from both specific pathogen-free and conventional swine. NPTr epithelial cells and AMs were inoculated with either culture medium, swIAV at a MOI of 0.5, PRRSV at a MOI of 1, or both viruses simultaneously. Samples were collected at 1, 12, 24, and 48 h. On the left side are presented the results for SPF pig AMs and on the right are the results for conventional pig AMs. **(A–H)** The expression of the antiviral genes IFNα, IFNβ, MX2, and PKR were analyzed by RT-qPCR. Statistics Kruskal-Walis, non-parametric test. Different letters (a–d) indicate that the considered group (specified by its color) was significantly different from the Mock group (a), from the swIAV group (b), from the PRRSV group (c) or from the swIAV/PRRSV group (d) with *p* < 0.05 (mean ± SD; *n* = 6 for SPF swine AMs; *n* = 4 for conventional swine AMs). Data are combined from 2 independent experiments for AMs originating from SPF or conventional swine.

A co-culture system involving AMs and NPTr cells was established. The objective of using such a system was to study the interaction between swIAV and PRRSV infections in the presence of their respective main target cells. Both viral infections were compared in co-cultures using AMs derived either from conventional or SPF pigs (Figures 4, 5).

In NPTr/AMs co-culture, similar patterns were observed in the expression of the swIAV and PRRSV genomes in cell lysates, whatever the AMs origin, i.e., from SPF or conventional pigs. No significant (p > 0.05) difference in swIAV genome replication was observed within the cells, whether through single inoculation or co-inoculation with PRRSV (Figures 4A,B). However, we observed complete inhibition of PRRSV replication as early as 12 hpi when co-inoculated with swIAV in co-culture with SPF pig AMs, and as early as 24 hpi in co-culture with conventional pig AMs (Figures 4C,D; Supplementary Figures S4). The inhibition was significantly more pronounced at both 24 hpi (*p* < 0.01) and 48 hpi (*p* < 0.05) compared to that measured after PRRSV inoculation in monoculture of SPF pig AMs (Supplementary Figures S5A,B). Additionally, the percentage of PRRSV inhibition was significantly higher in co-cultures making use of SPF AMs compared to conventional AMs at 24 h (*p* < 0.05), but not at 48 h (Supplementary Figures S5C,D). In co-inoculated AMs from conventional pigs, PRRSV replication was inhibited in all animals, including animals *A* and *B*, which were initially used for monoculture experiments and for which no inhibition was observed (Supplementary Figure S4D). Detection of PRRSV in supernatants followed similar patterns than intracellular virus detection, with swIAV-mediated inhibition (Supplementary Figure S6).

Regarding the expression of antiviral genes during co-culture (Figures 5A–H), significant (*p* < 0.05) expression of IFNα was observed at an early stage (12 h) post-inoculation with swIAV alone, whatever the type of AMs used in the co-culture (Figures 5A,B). For IFNβ, significant early expression at 12 hpi was also observed in the swIAV (*p* < 0.05) and swIAV/PRRSV (*p* < 0.01) conditions in co-culture using AMs from SPF pigs, whereas in the PRRSV condition, IFNβ expression occurred with a slight delay at 24 hpi (*p* < 0.01) (Figure 5C). In co-cultures with AMs from conventional pigs, changes in IFNβ expression showed similar trend to those observed in co-cultures with AMs from SPF pigs, with increased expression at 12 hpi. However, the results were not significant compared to the Mock group (*p* > 0.05) in both the swIAV (*p* = 0.09) and swIAV/PRRSV (*p* = 0.06) conditions (Figure 5D).

Regarding ISG expression, in co-culture with SPF pig AMs, the MX2 gene showed an early increase at 12 hpi in the swIAV and swIAV/PRRSV conditions (*p* < 0.05), persisting at high levels up to 48 hpi (*p* < 0.05). In the PRRSV group, significant (*p* < 0.05) MX2 expression was only observed from 48 hpi onwards (Figure 5E). The expression pattern of MX2 at 12 hpi with conventional AMs in the swIAV and swIAV/PRRSV conditions was similar to that in co-cultures with SPF pig AMs. However, these results were not significant compared to the Mock group (*p* = 0.10) for both swIAV and swIAV/PRRSV condition (Figure 5F).

A significant difference in expression of the PKR gene was found with SPF AMs at 24 hpi for the swIAV condition (*p* < 0.01) and at 48 hpi for the PRRSV condition (*p* < 0.01), as compared to mock-inoculated cells (Figure 5G). In contrast, the co-cultures with AMs from conventional pigs also exhibited a significant (*p* < 0.05) increase in PKR expression for the swIAV/PRRSV condition, from 12 to 48 hpi (Figure 5H), despite the non-significance of IFNβ expression (Figure 5D).

## 4 Discussion

AMs are among the first cells to respond to IAV infection due to their strategic location in the respiratory tract. They play a crucial role in fighting infection, as evidenced by studies showing that AMs-depleted animals exhibit high pulmonary virus replication, increased tissue damage, and elevated mortality rates (46, 47).

Despite the known importance of AMs in IAV infection outcomes and pathogenicity, the impact of AMs infection by IAV remains debated in the literature. Some studies have shown active IAV replication in AMs, while others have reported non-productive replication (26, 48). Research on IAV replication in human and murine AMs has revealed productive viral replication after inoculation of highly pathogenic avian influenza virus (H5N1) but not of human seasonal strains (26, 48). Additionally, it has been demonstrated that human monocyte-derived macrophages, but not AMs, can support productive replication of some human and avian IAV subtypes (26, 49).

Concerning porcine macrophages, previous studies have shown that the human H1N1pdm09 virus can infect and replicate in a porcine macrophage cell line (3D4), leading to apoptosis (21). However, in an older study, infection of porcine AMs *in vitro* with seasonal human strains H3N2 or H1N1 did not show apoptosis after 48 h, despite viral infection of the cells. Additionally, *in vivo*, AMs obtained from pigs inoculated with the same strains also did not demonstrate apoptosis 5 days post-infection (50). Furthermore, porcine AMs have been shown to be susceptible to various strains of avian, human, and swine IAVs, with only avian strains inducing apoptosis 6 h post-inoculation (22).

Here, we examined the effects of swIAV on porcine AMs. We observed that the tested H1_hu_N2 swIAV strain led to non-productive infection of AMs and induced their death only at a high multiplicity of infection (MOI 2).

This variability observed in the impact of different IAV strains on macrophages seems to be influenced by the macrophage subtype, the species used, and the IAV genetic factors. For instance, an amino acid replacement in the virus hemagglutinin might alter the IAV infection and cell death susceptibility of AMs. Cardenas et al. (51) demonstrated this with an H3N2 reassortant (hVIC/11) containing the HA and NA segments from a seasonal human virus and the other genes from a swine strain (sOH/04). This combination, including a point mutation near the HA receptor binding site (A138S), showed a higher affinity for porcine AMs. The hVIC/11 A138S infected both the upper and lower respiratory tracts, whereas the hVIC/11 was only detected in the upper respiratory tract 5 days post-infection. Additionally, AMs in bronchoalveolar lavage fluid samples were significantly reduced in pigs infected with sOH/04 and hVIC/11 A138S, but not in those infected with hVIC/11. Further analysis using the porcine AMs cell line 3D4/21 showed that the A138S mutation increases the virus’ replication capacity compared to hVIC/11.

Our understanding of abortive IAV infection in macrophages is complicated by the diversity of macrophage sources (such as those derived from mice or humans) and the various IAV strains, including highly pathogenic ones. Studies aimed at understanding the mechanisms of viral abortion in macrophages have revealed that infection of murine and human macrophages by the seasonal H1N1 virus can be blocked at different stages: (i) during the viral entry phase (52), (ii) after internalization but before nuclear entry (48), (iii) at a late stage of the viral replication, during the assembly of viral particles, preventing the release of new virions (52, 53). Additionally, cell death mechanisms, such as apoptosis and necroptosis, can also limit the spread of the virus. Indeed, IAV infection can induce macrophage death (22, 54), as observed in our results at a high MOI of 2, although our experimental co-inoculation conditions were set up in order to avoid this IAV-mediated cell-death.

In our study, we found that swIAV replication in AMs was not modulated in the presence of PRRSV. By contrast, the presence of swIAV impacted PRRSV replication, both in AMs monoculture and in NPTr/AMs co-culture. This phenomenon was accompanied by an early induction of IFN-I and ISG, as observed at 12 hpi. Since IAV is known to induce IFN-I production in AMs (15, 21, 55), the increased IFN-I and ISG transcript levels might be accountable for IFN-mediated inhibition of PRRSV replication in agreement with a previous *in vitro* work studying swIAV/PRRSV-2 strains interferences (33).

The induction of IFN-I by swIAV and the subsequent inhibition of PRRSV are consistent with previous *in vivo* studies, as we showed that swIAV infection significantly affected PRRSV multiplication in SPF pig lungs (17). Correlation studies revealed an important role of IFNα in the interference between these two viral infections. Another *in vivo* study in SPF pigs demonstrated that swIAV infection delayed the viremia of a live attenuated PRRSV vaccine, with an early an IFNα increase detected after swIAV infection, likely responsible for PRRSV replication inhibition (56). Additionally, this local upregulation of IFNα might account for the increased anti-PRRSV CD8 T-cell response observed *in vivo* during swIAV H3N2/PRRSV-2 co-infections (31).

PRRSV has developed several strategies to evade IFN action and to persist in the host (10). For example, it can increase specific porcine miRNAs like let-7b, miR-26a, miR-34a and miR-145. These miRNAs can hinder the expression of IFNβ protein in primary AMs by directly targeting nucleic acid sequences in the 3′UTR of porcine IFNβ mRNA. This action suppresses IFNβ protein expression at the post-transcriptional level (57). This may explain why PRRSV was not inhibited when inoculated alone on AMs and NPTr/AMs cultures, despite strong IFN-I mRNA expression as early as 24 hpi. The increase in IFNβ transcript expression did not lead to ISGs upregulation, suggesting an absence of functional IFNβ protein.

In swIAV and swIAV/PRRSV co-inoculation, we observed a type-I IFN transcriptomic induction triggered by IAV, lower but earlier than upon PRRSV inoculation. This transcriptomic expression is paralleled with ISG induction, in agreement with an IFN-I effective protein expression. This IAV mediated antiviral gene expression correlates with the inhibition of PRRSV replication.

Interestingly, the use of conventional pig AMs revealed diversity in host responses to viruses. AMs from animals *A*, *B*, and *C* reacted differently from those of animal *D* and SPF pig AMs in monoculture. As mentioned earlier, PRRSV inhibition in animal *D* and SPF pig AMs would be attributed to IFN-I and ISG induction by AMs. However, in AMs from animals *A*, *B*, and *C*, there was no ISG expression, suggesting an absence of IFN-I production, which might allow PRRSV replication.

In our study, we used either AMs collected from 5-to 6-month-old conventional pigs from INRAE controlled breeding, or AMs from SPF pigs sampled at 2 months of age. More than the age, the sanitary status seemed to be the most important factor playing a role in the differences we observed following swIAV/PRRSV co-inoculations. Indeed, although the four conventional pigs were older than SPF pigs, one of them showed a response similar to SPF AMs.

More specifically, we did not observe differences in PRRSV replication levels in the PRRSV single-inoculated condition, whether the AMs were from older or younger pigs.

Interestingly, an *in vitro* study has shown that pulmonary intravascular macrophages (PIMs) from 4-week-old pigs yielded a higher virus titer following PRRSV infection compared to PIMs from 4-month-old pigs (58). However, these differences were not observed in our *in vitro* data, indicating that the age of the pigs does not have a significant impact on PRRSV replication in AMs under our experimental conditions.

In summary, although age-related susceptibility variations have been previously noted, our *in vitro* results do not show such differences, suggesting that sanitary status is a more important determinant for the response of AMs to PRRSV infection.

ANSES’ SPF animals were maintained in a highly clean environment and were free of all respiratory pathogens, as they were reared under air filtration, while INRAE’s animals came from a controlled environment (59) not deprived of potential respiratory pathogens.

Interestingly a recent study highlighted the differential viral replication and immune responses of SPF and farm-raised Large White domestic pigs upon African swine fever systemic infections (60). At steady state, SPF pigs exhibited a less inflammatory and antiviral basal whole blood transcriptomic profile compared to farm-raised pigs. We indeed observed a similar trend with type-I IFN and ISG lower expressions in AM from SPF compared to conventional pigs (data not shown).

Observations in mice have shown that following recovery from initial pneumonia, murine AMs exhibited modified phagocytic activity for several weeks (45). These paralyzed AMs originated from resident AMs that underwent a tolerogenic epigenetic training program. This may explain why, in our study, AMs from 1 out of 4 conventional animals showed similar behavior to AMs from SPF pigs, suggesting that the other three conventional pigs might have been infected by other pathogens including swIAV during their early lifetime.

Another possibility described in mice is the depletion of fetal liver-origin AMs upon respiratory infections, leading to their replacement by monocyte-derived AMs (61, 62). These monocyte-derived AMs increased IAV-induced pathogenicity and mortality. This study also showed that the origin of AMs, rather than their previous experience, could determine their long-term function in recurrent viral infections (63). Thus, in some physiological conditions, the origin of AMs, rather than their previous experience, could determine their long-term function, although this hypothesis remains difficult to explore in pigs.

The results presented here might suggest that AMs from most of conventional pigs (Animals *A*, *B*, and *C*) have undergone modifications and have been influenced by external factors at some point in their lives.

In NPTr/AMs co-cultures, regardless of whether the AMs came from conventional or SPF pigs, both sources showed similar ability to inhibit PRRSV replication in the PRRSV/swAIV co-inoculation conditions. However, a greater inhibition of PRRSV was observed in co-culture with SPF AMs at 24 h only and not at 48 h. This inhibition could be attributed to swIAV replication in NPTr cells, leading to viral particle release entering and depleting AMs, but also to the production of IFN produced by AMs and by epithelial cells following swIAV infection.

## 5 Conclusion

This study showed that, despite limited replication in AMs, swIAV could inhibit PRRSV replication in porcine AMs, likely through IFN-I modulation. We also observed variations in this swIAV-mediated PRRSV inhibition depending on the origins of the AMs. Whether these variations are due to the animal’s health history or to another unidentified parameter remain to be determined. Since AMs are targets for many infections and have a long lifespan, these observations justify the need for further research on the long-term impact of infections on innate respiratory immunity, as well as the study of viral interactions within the context of the swine respiratory disease complex.

Notably, our co-culture experiments revealed that the presence of respiratory tracheal epithelial cells significantly enhances the swIAV-induced inhibition of PRRSV replication, indicating a synergistic effect between AMs and epithelial cells in antiviral responses, regardless of whether the AMs are derived from SPF or conventional pigs.

Understanding these interactions could lead to the development of more effective prevention and control strategies for respiratory diseases in pigs, ultimately improving animal health and reducing economic losses in the swine industry.

## Data Availability

The datasets presented in this study can be found in online repositories. The names of the repository/repositories and accession number(s) can be found at: https://entrepot.recherche.data.gouv.fr/dataset.xhtml?persistentId=doi:10.57745/NW7QBV, https://doi.org/10.57745/NW7QBV.
