# Supplementary material for: Impact of swine influenza A virus on porcine reproductive and respiratory syndrome virus infection in alveolar macrophages
Source: Front Vet Sci. 2024 Aug 26;11:1454762. doi: 10.3389/fvets.2024.1454762 (PMC11381391; doi:10.3389/fvets.2024.1454762)
Supplement: Supplementary file 1 [file Presentation_1.pptx]

## Slide 1
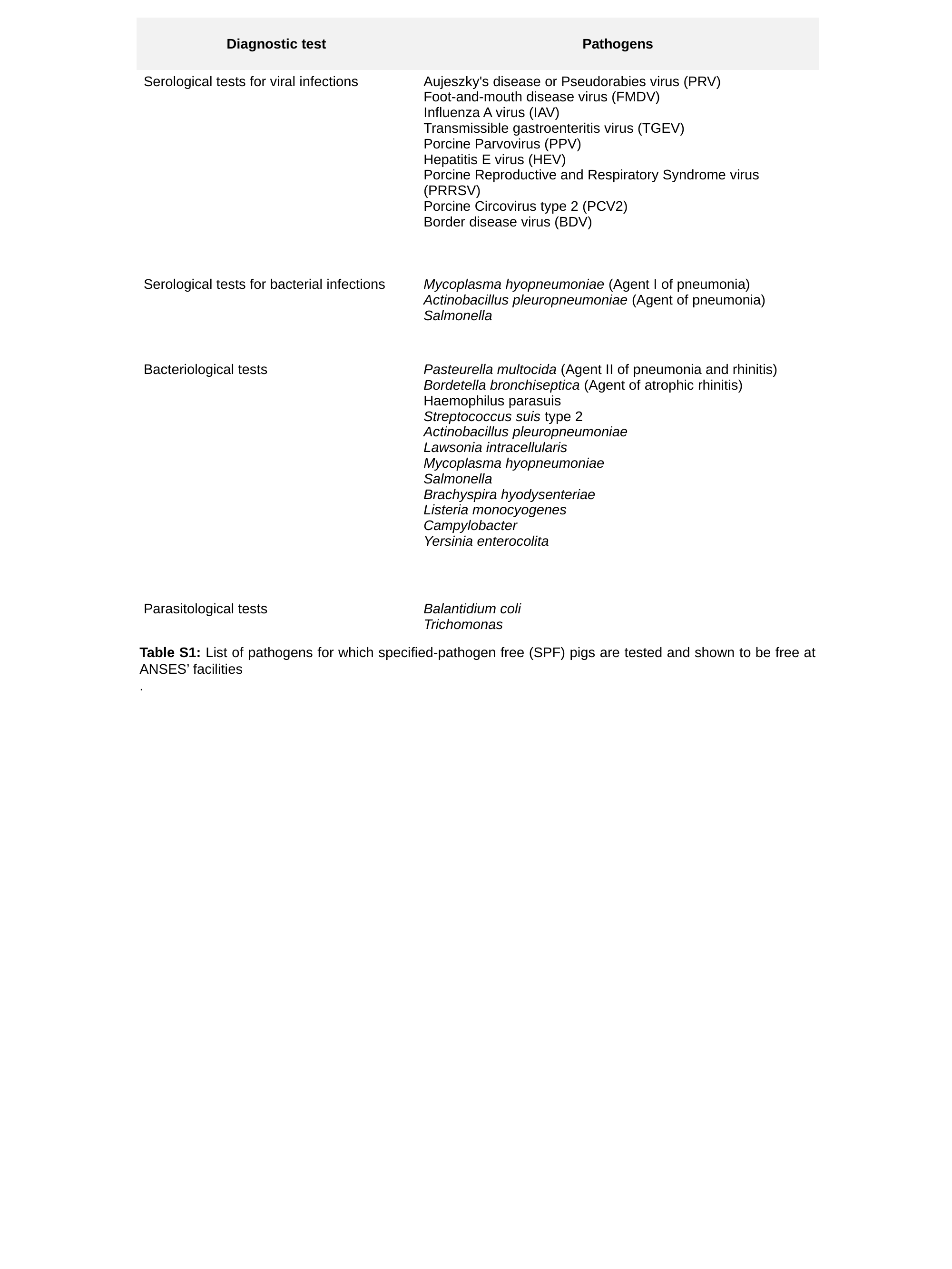

| Diagnostic test | Pathogens |
| --- | --- |
| Serological tests for viral infections | Aujeszky's disease or Pseudorabies virus (PRV) Foot-and-mouth disease virus (FMDV) Influenza A virus (IAV) Transmissible gastroenteritis virus (TGEV) Porcine Parvovirus (PPV) Hepatitis E virus (HEV) Porcine Reproductive and Respiratory Syndrome virus (PRRSV) Porcine Circovirus type 2 (PCV2) Border disease virus (BDV) |
| Serological tests for bacterial infections | Mycoplasma hyopneumoniae (Agent I of pneumonia) Actinobacillus pleuropneumoniae (Agent of pneumonia) Salmonella |
| Bacteriological tests | Pasteurella multocida (Agent II of pneumonia and rhinitis) Bordetella bronchiseptica (Agent of atrophic rhinitis) Haemophilus parasuis Streptococcus suis type 2 Actinobacillus pleuropneumoniae Lawsonia intracellularis Mycoplasma hyopneumoniae Salmonella Brachyspira hyodysenteriae Listeria monocyogenes Campylobacter Yersinia enterocolita |
| Parasitological tests | Balantidium coli Trichomonas |
Table S1: List of pathogens for which specified-pathogen free (SPF) pigs are tested and shown to be free at ANSES’ facilities
.

## Slide 2
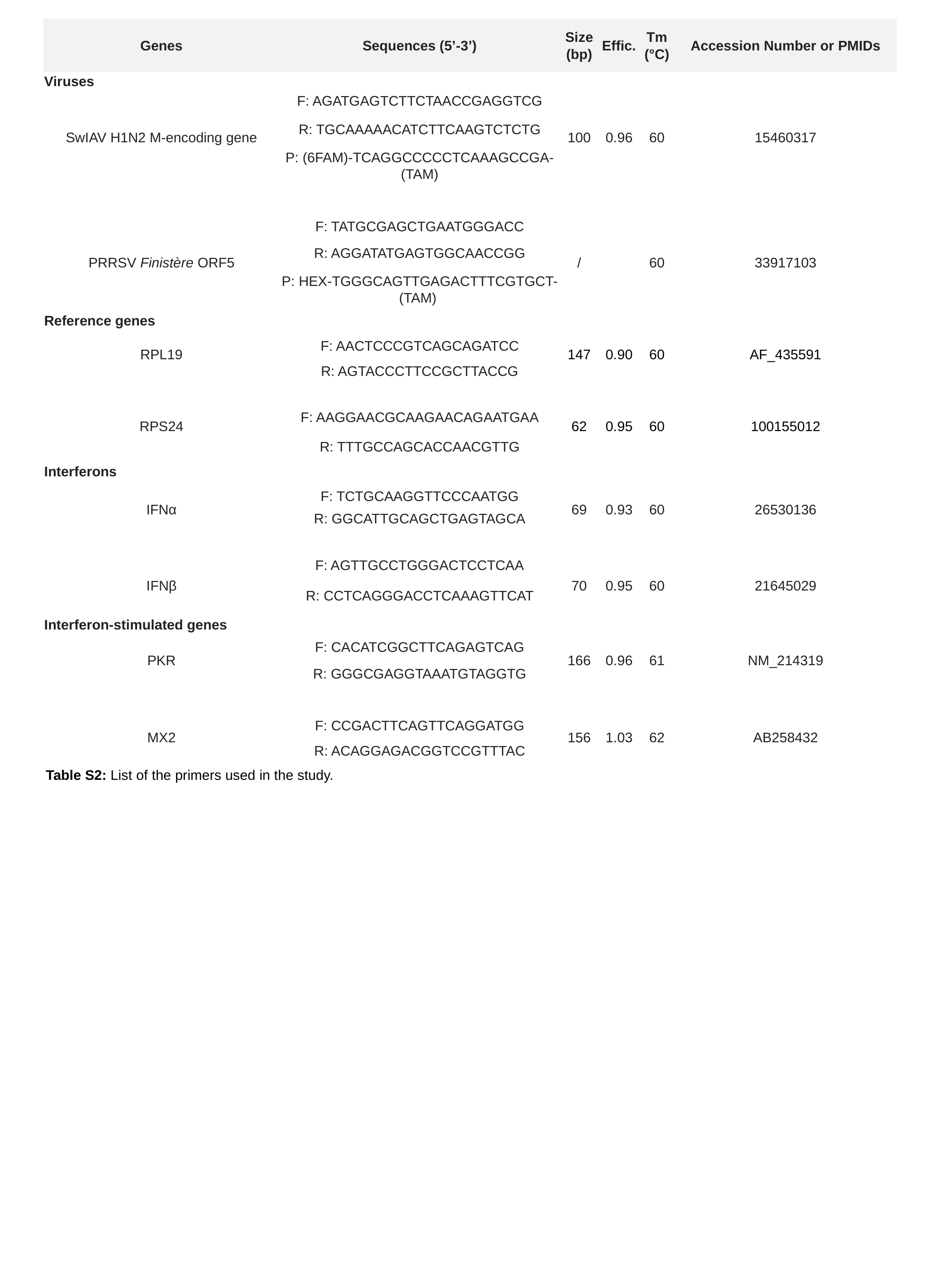

| Genes | Sequences (5’-3’) | Size (bp) | Effic. | Tm (°C) | Accession Number or PMIDs |
| --- | --- | --- | --- | --- | --- |
| Viruses | | | | | |
| SwIAV H1N2 M-encoding gene | F: AGATGAGTCTTCTAACCGAGGTCG | 100 | 0.96 | 60 | 15460317 |
| | R: TGCAAAAACATCTTCAAGTCTCTG | | | | |
| | P: (6FAM)-TCAGGCCCCCTCAAAGCCGA-(TAM) | | | | |
| | | | | | |
| PRRSV Finistère ORF5 | F: TATGCGAGCTGAATGGGACC | / | | 60 | 33917103 |
| | R: AGGATATGAGTGGCAACCGG | | | | |
| | P: HEX-TGGGCAGTTGAGACTTTCGTGCT-(TAM) | | | | |
| Reference genes | | | | | |
| RPL19 | F: AACTCCCGTCAGCAGATCC | 147 | 0.90 | 60 | AF\_435591 |
| | R: AGTACCCTTCCGCTTACCG | | | | |
| | | | | | |
| RPS24 | F: AAGGAACGCAAGAACAGAATGAA | 62 | 0.95 | 60 | 100155012 |
| | R: TTTGCCAGCACCAACGTTG | | | | |
| Interferons | | | | | |
| IFNα | F: TCTGCAAGGTTCCCAATGG | 69 | 0.93 | 60 | 26530136 |
| | R: GGCATTGCAGCTGAGTAGCA | | | | |
| | | | | | |
| IFNβ | F: AGTTGCCTGGGACTCCTCAA | 70 | 0.95 | 60 | 21645029 |
| | R: CCTCAGGGACCTCAAAGTTCAT | | | | |
| Interferon-stimulated genes | | | | | |
| PKR | F: CACATCGGCTTCAGAGTCAG | 166 | 0.96 | 61 | NM\_214319 |
| | R: GGGCGAGGTAAATGTAGGTG | | | | |
| | | | | | |
| MX2 | F: CCGACTTCAGTTCAGGATGG | 156 | 1.03 | 62 | AB258432 |
| | R: ACAGGAGACGGTCCGTTTAC | | | | |
Table S2: List of the primers used in the study.

## Slide 3
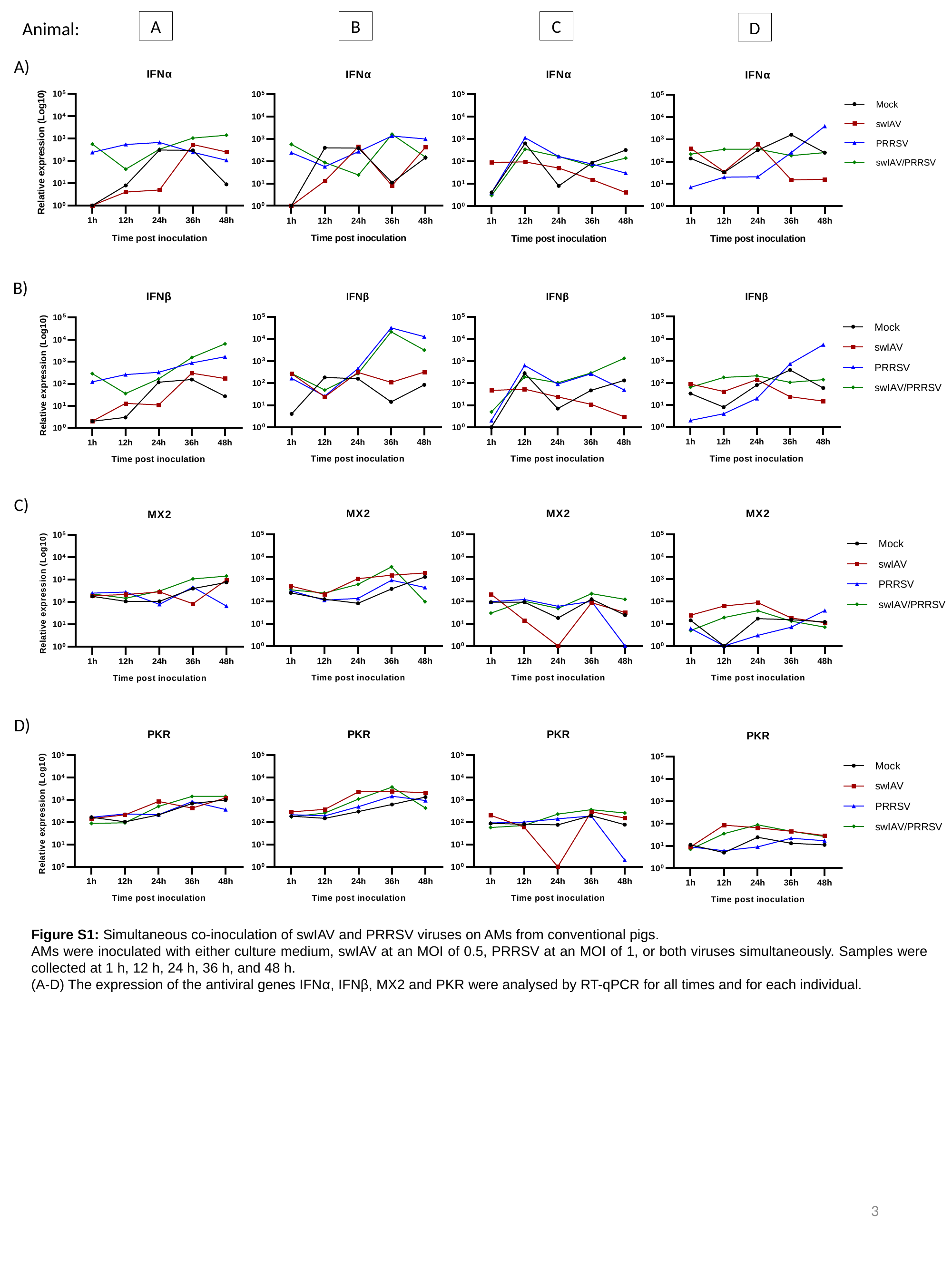

A
B
C
D
Animal:
A)
B)
C)
D)
Figure S1: Simultaneous co-inoculation of swIAV and PRRSV viruses on AMs from conventional pigs.
AMs were inoculated with either culture medium, swIAV at an MOI of 0.5, PRRSV at an MOI of 1, or both viruses simultaneously. Samples were collected at 1 h, 12 h, 24 h, 36 h, and 48 h.
(A-D) The expression of the antiviral genes IFNα, IFNβ, MX2 and PKR were analysed by RT-qPCR for all times and for each individual.
3

## Slide 4
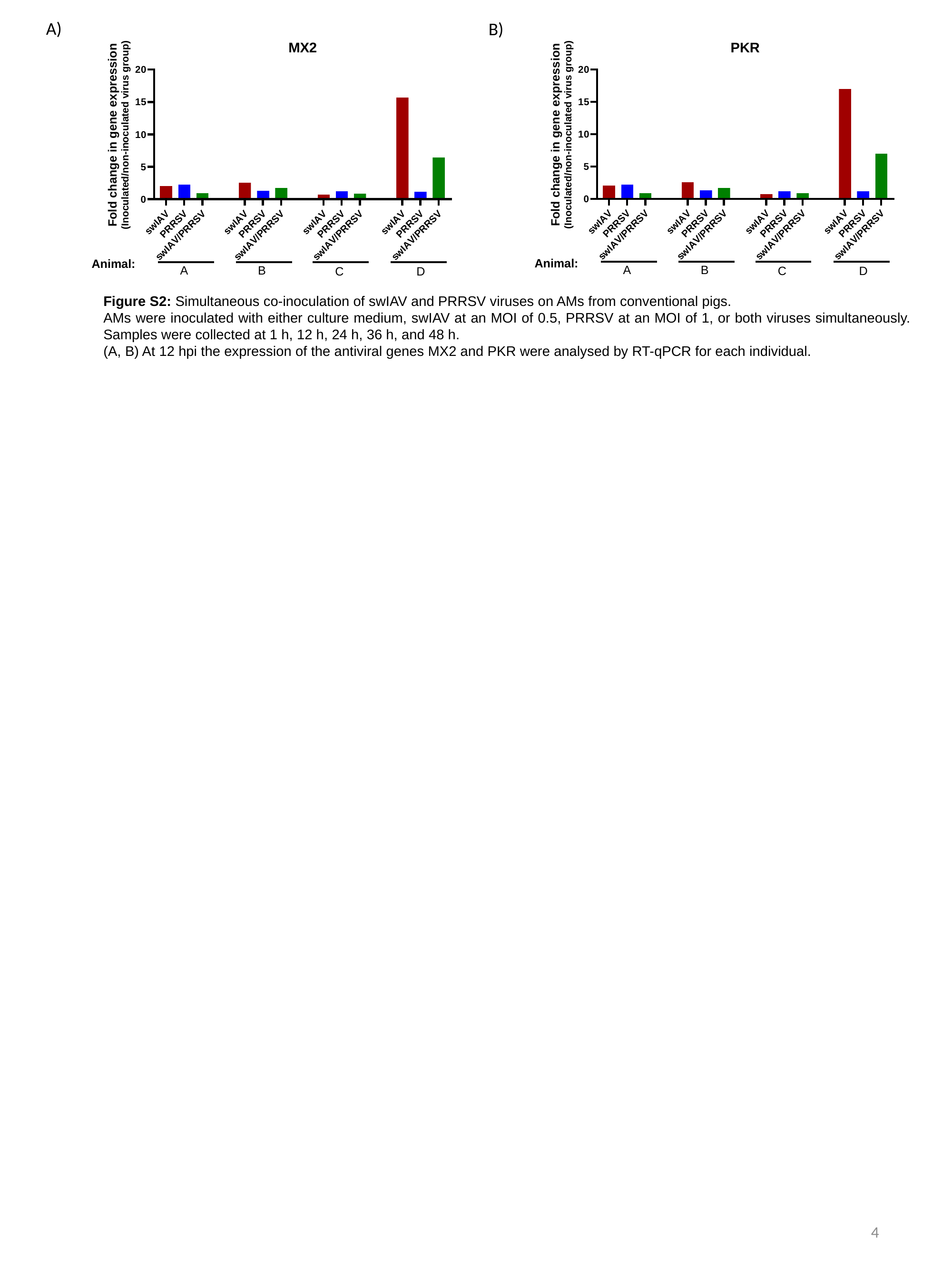

A)
B)
Figure S2: Simultaneous co-inoculation of swIAV and PRRSV viruses on AMs from conventional pigs.
AMs were inoculated with either culture medium, swIAV at an MOI of 0.5, PRRSV at an MOI of 1, or both viruses simultaneously. Samples were collected at 1 h, 12 h, 24 h, 36 h, and 48 h.
(A, B) At 12 hpi the expression of the antiviral genes MX2 and PKR were analysed by RT-qPCR for each individual.
4

## Slide 5
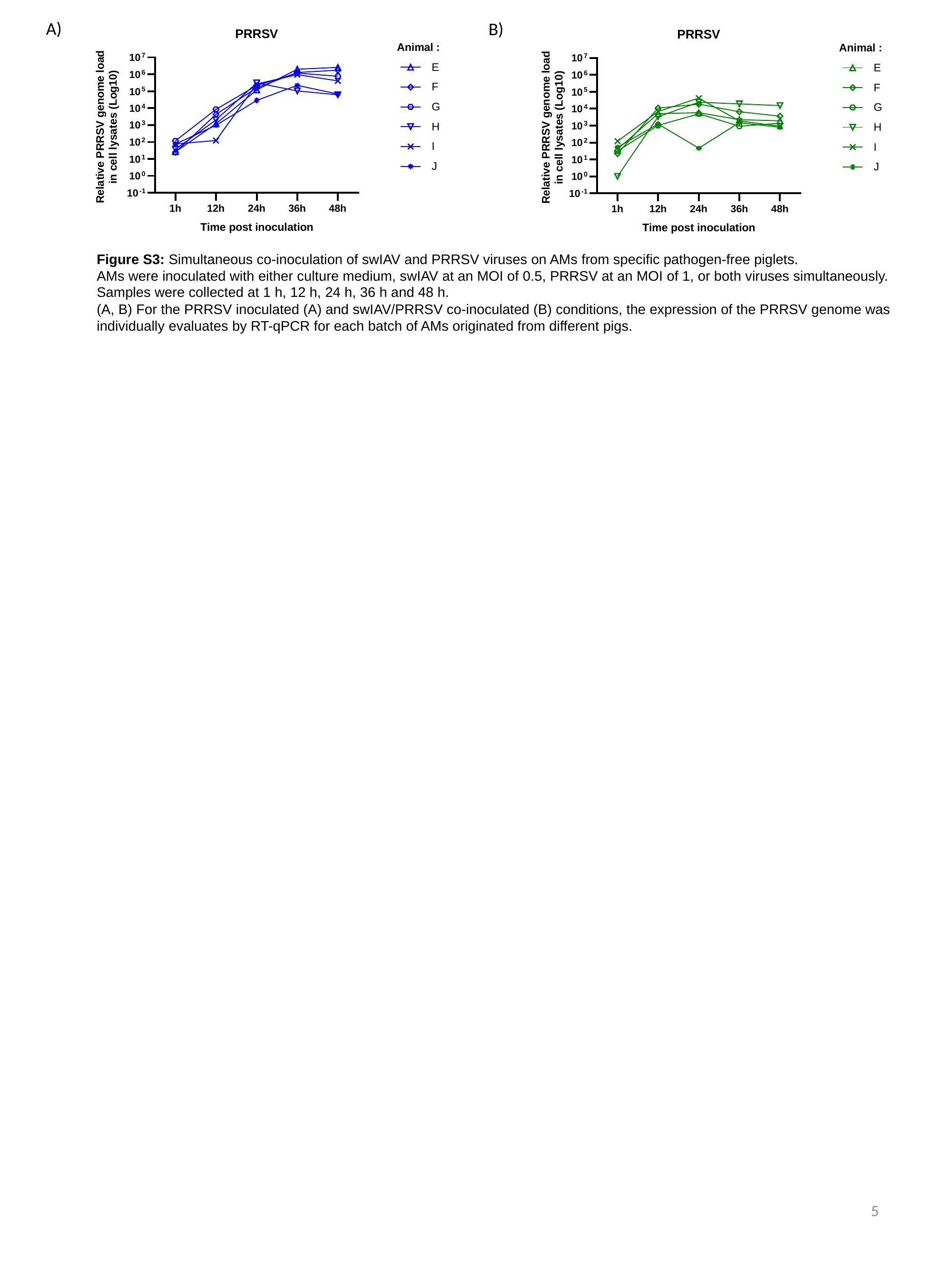

A)
B)
Figure S3: Simultaneous co-inoculation of swIAV and PRRSV viruses on AMs from specific pathogen-free piglets.
AMs were inoculated with either culture medium, swIAV at an MOI of 0.5, PRRSV at an MOI of 1, or both viruses simultaneously. Samples were collected at 1 h, 12 h, 24 h, 36 h and 48 h.
(A, B) For the PRRSV inoculated (A) and swIAV/PRRSV co-inoculated (B) conditions, the expression of the PRRSV genome was individually evaluates by RT-qPCR for each batch of AMs originated from different pigs.
5

## Slide 6
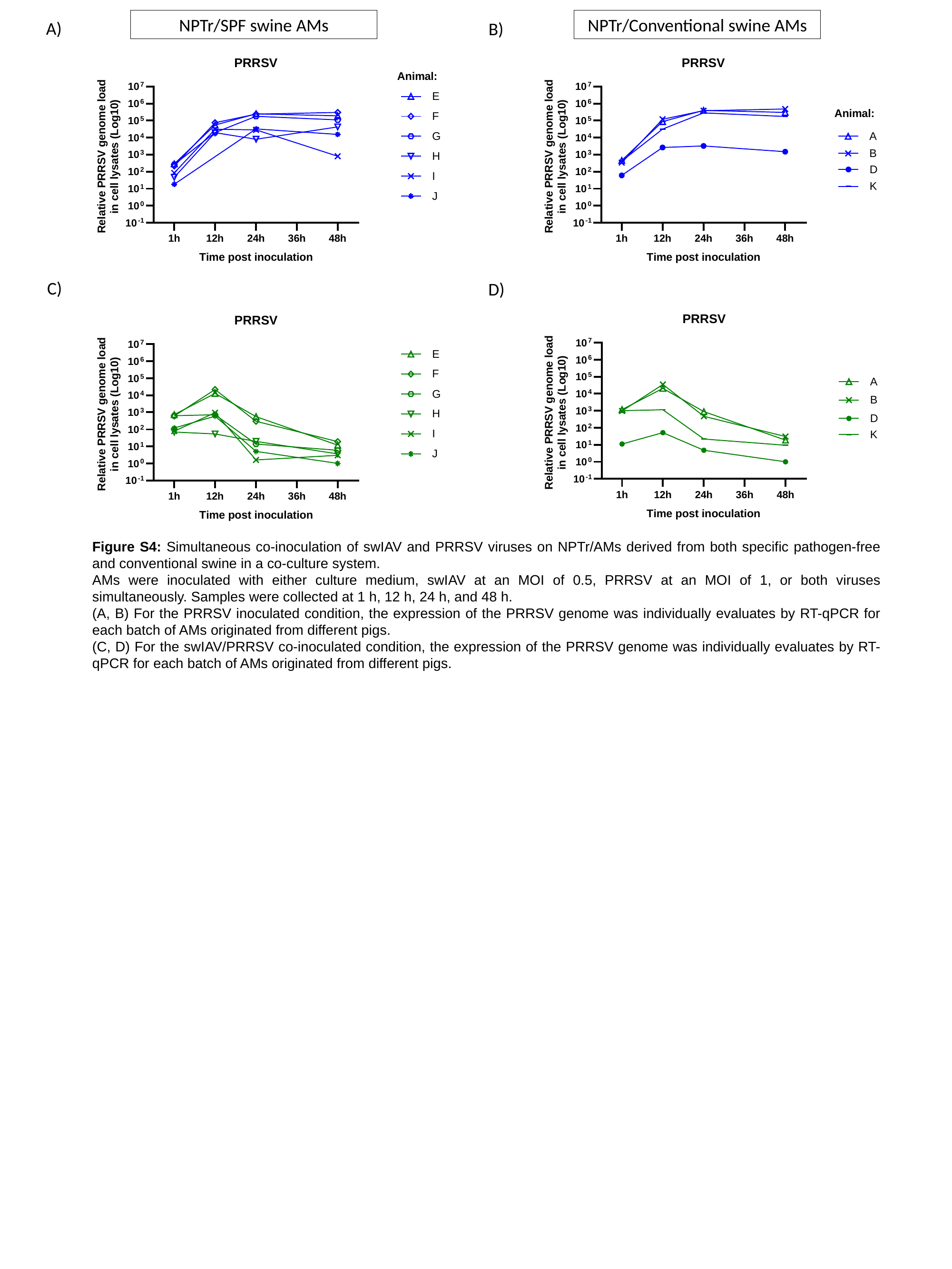

NPTr/SPF swine AMs
NPTr/Conventional swine AMs
A)
B)
C)
D)
Figure S4: Simultaneous co-inoculation of swIAV and PRRSV viruses on NPTr/AMs derived from both specific pathogen-free and conventional swine in a co-culture system.
AMs were inoculated with either culture medium, swIAV at an MOI of 0.5, PRRSV at an MOI of 1, or both viruses simultaneously. Samples were collected at 1 h, 12 h, 24 h, and 48 h.
(A, B) For the PRRSV inoculated condition, the expression of the PRRSV genome was individually evaluates by RT-qPCR for each batch of AMs originated from different pigs.
(C, D) For the swIAV/PRRSV co-inoculated condition, the expression of the PRRSV genome was individually evaluates by RT-qPCR for each batch of AMs originated from different pigs.
6

## Slide 7
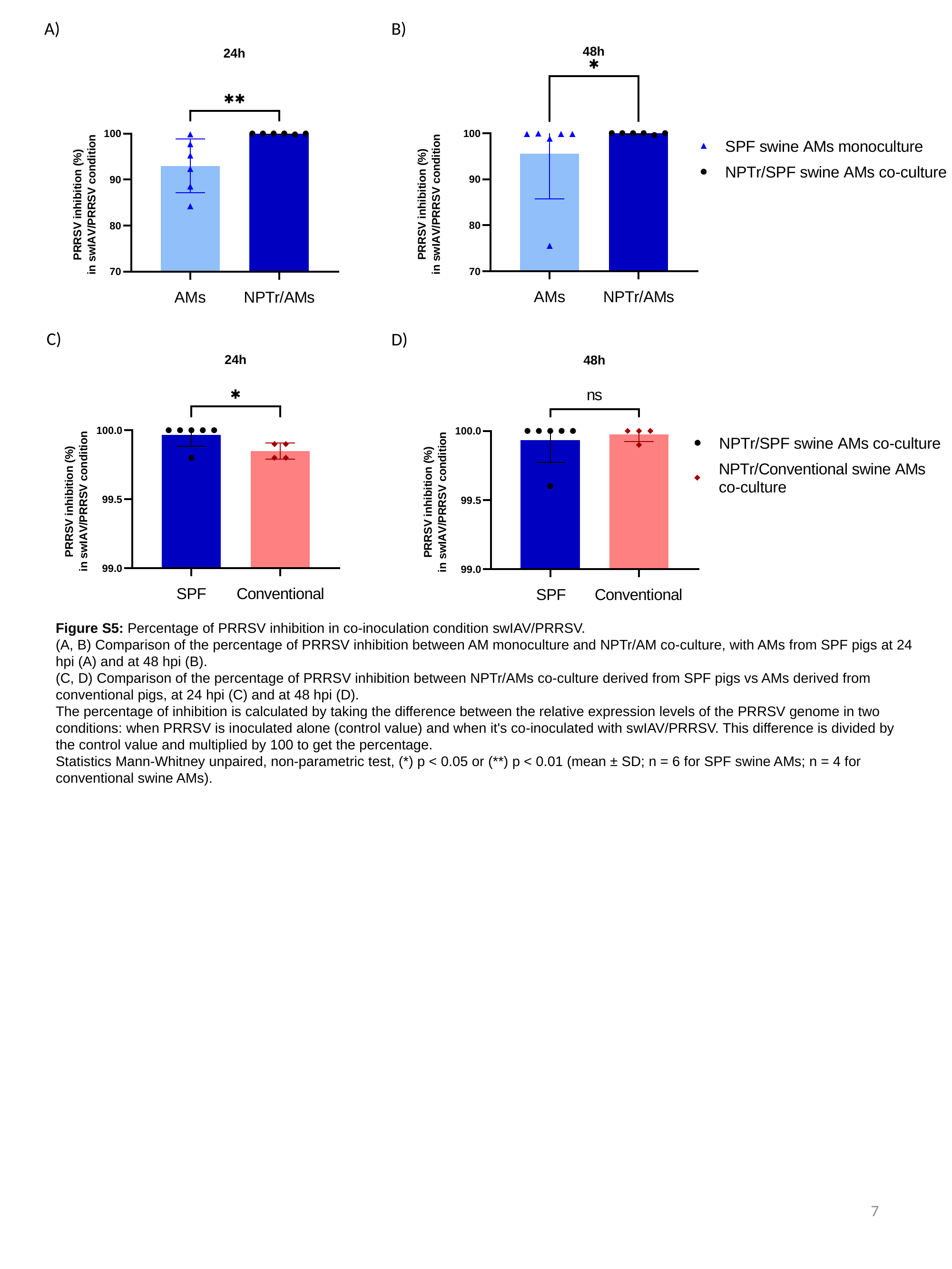

A)
B)
C)
D)
Figure S5: Percentage of PRRSV inhibition in co-inoculation condition swIAV/PRRSV.
(A, B) Comparison of the percentage of PRRSV inhibition between AM monoculture and NPTr/AM co-culture, with AMs from SPF pigs at 24 hpi (A) and at 48 hpi (B).
(C, D) Comparison of the percentage of PRRSV inhibition between NPTr/AMs co-culture derived from SPF pigs vs AMs derived from conventional pigs, at 24 hpi (C) and at 48 hpi (D).
The percentage of inhibition is calculated by taking the difference between the relative expression levels of the PRRSV genome in two conditions: when PRRSV is inoculated alone (control value) and when it's co-inoculated with swIAV/PRRSV. This difference is divided by the control value and multiplied by 100 to get the percentage.
Statistics Mann-Whitney unpaired, non-parametric test, (*) p < 0.05 or (**) p < 0.01 (mean ± SD; n = 6 for SPF swine AMs; n = 4 for conventional swine AMs).
7

## Slide 8
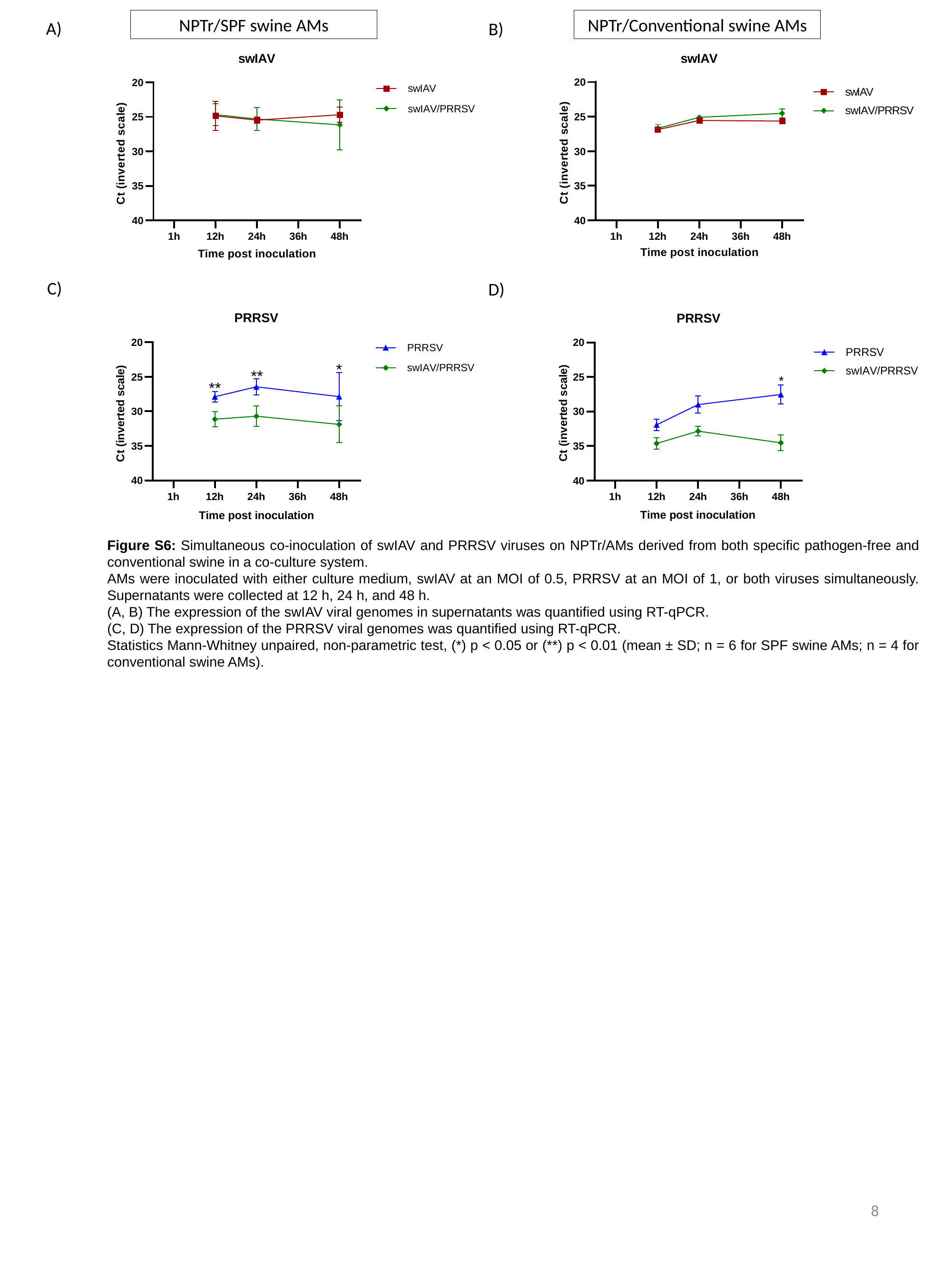

NPTr/SPF swine AMs
NPTr/Conventional swine AMs
A)
B)
C)
D)
Figure S6: Simultaneous co-inoculation of swIAV and PRRSV viruses on NPTr/AMs derived from both specific pathogen-free and conventional swine in a co-culture system.
AMs were inoculated with either culture medium, swIAV at an MOI of 0.5, PRRSV at an MOI of 1, or both viruses simultaneously. Supernatants were collected at 12 h, 24 h, and 48 h.
(A, B) The expression of the swIAV viral genomes in supernatants was quantified using RT-qPCR.
(C, D) The expression of the PRRSV viral genomes was quantified using RT-qPCR.
Statistics Mann-Whitney unpaired, non-parametric test, (*) p < 0.05 or (**) p < 0.01 (mean ± SD; n = 6 for SPF swine AMs; n = 4 for conventional swine AMs).
8
